# SplenoMegaly study (SMS): exploring the etiologies for “unexplained” splenomegalies in the real world

**DOI:** 10.1186/s13023-025-03768-3

**Published:** 2025-06-06

**Authors:** Guillaume Denis, Louis Terriou, Thomas Sené, Regis Costello, Martin Michaud, Audrey Lagadec, Frédéric Bauduer, Laurence Sanhes, Christian Rose, Geoffrey Urbanski, Marc G. Berger

**Affiliations:** 1 Department of Internal Medicine and Hematology, Rochefort Hospital, Rochefort, France; 2 Department of Internal Medicine and Immunology, Centre de Référence des Maladies Autoimmunes Systémiques Rares du Nord et Nord-Ouest de France (CeRAINO), CHRU, Lille, France; 3 Department of Internal Medicine, Clinique Axium, Aix En Provence, France; 4 Department of Hematology and Cellular Therapy, La Conception Hospital, Marseille, France; 5 Department of Internal Medicine, Joseph Ducuing Hospital, Toulouse, France; 6 Medical Department, Sanofi, Gentilly, France; 7 Department of Hematology, Côte Basque Hospital, Bayonne, France; 8 College of Health Sciences, University of Bordeaux, Bordeaux, France; 9 Department of Hematology, Saint Jean Hospital, Perpignan, France; 10 Department of Oncology and Hematology, Saint Vincent de Paul Hospital, Lille, France; 11 Department of Internal Medicine and Clinical Immunology, University Hospital Angers, Angers, France; 12 Department of Orofacial Sciences, School of Dentistry, University of California, San Francisco, CA USA; 13 Department of Immunology and Allergology, Hôpitaux Universitaires de Genève, Geneva, Switzerland; 14 Department of Biological Hematology and EA 7453 CHELTER, University Hospital of Clermont-Ferrand and Université Clermont Auvergne, Clermont-Ferrand, France

## Abstract

**Background:**

The predominant etiologies of splenomegaly (SM) are readily discernible through routine clinical assessments, yet in numerous instances, the etiological basis remains elusive. Subsequent diagnostic steps are not consensual and are challenging for physicians due to miscellaneous causes and non-specific symptoms. This study aimed to estimate the prevalence of Gaucher disease (GD) and other etiologies in patients presenting with unexplained splenomegaly (SM) after exclusion of first intention-diagnoses (e.g., portal hypertension, hematological malignancy, hemolytic anemia, and infection) based on basic physical examination, patient interview, and routine biological exams (e.g., full blood count, liver enzymes, and reticulocyte count). Additionally, the study aimed to describe the diagnostic tests performed and the most frequent associations observed. This French prospective, observational, multicenter, longitudinal SMS study enrolled 505 patients from September 2015 to April 2020, aged ≥ 15 years, referred to hematology or internal medicine departments, with a diagnostically confirmed SM (spleen length ≥ 13 cm). SM was defined as unexplained when routine clinical and biological tests were negative. Patients were followed up until an etiology was identified or up to 18 months after inclusion.

**Results:**

An etiology of SM was found in 223 (44.5%) of 501 patients with follow up. Patients with explained SM were older, had a larger spleen, and had altered biological parameters compared with patients with unexplained SM. There was a higher prevalence of non-malignant diseases than hematological malignancies (27.1% vs. 17.0%). Overall, lysosomal storage diseases (LSDs) were diagnosed in 10 patients (2.0%), including 4 patients with GD (0.8%).

**Conclusions:**

A list of potential predictive factors for the main diagnostic categories was identified that could optimize the diagnostic strategy for unexplained SM. This study provides new insights into exploring SM in the real world and proposes clinical and biological factors associated with specific etiologies.

**Clinical trial registration:**

NCT04430881.

**Supplementary Information:**

The online version contains supplementary material available at 10.1186/s13023-025-03768-3.

## Background

Splenomegaly (SM) is defined as an abnormal enlargement of the spleen. SM is a rare condition in the general population, with an estimated incidence of 0.3% of admissions in United States (US) hospitals [1] and 2% of the total US population [2]. In clinical practice, it could be discovered during a physical examination or in an imaging setting, such as ultrasound or computerized tomography (CT). Defining SM remains a source of debate, due to variability in the shape and size of the spleen according to physiological conditions, height, sex, age, and ethnic origin [3]; however, it is commonly accepted that a clearly palpable SM and/or a maximal length of ≥ 13 cm determined by radiological investigation could be considered as a criterion to define SM [4, 5]. 

The mechanism of splenic enlargement varies depending on the underlying cause, of which the most frequently reported in previous studies are hematological (16–66%), liver (9–41%), and infectious diseases (9–36%) in Europe and the US [1, 6, 7]. In 4–10% of patients, congestive or inflammatory diseases are observed, while primary splenic diseases are reported in 1–6% of cases. One to eight% of cases remain idiopathic or are associated with other underlying conditions. However, for cases of SM where no obvious etiology is found through questioning, clinical examination, and basic investigation, other diagnoses need to be investigated.

As no recommendations on the basic exploratory workup or the progression of complementary examinations have been established, the evaluation strategy is particularly influenced by the specialization of the physicians taking care of the patient. Given the large spectrum of underlying diseases sharing several signs and symptoms, differential diagnosis typically requires stepwise investigations, including a patient interview, a clinical examination, and routine biological tests [8, 9, 10]. 

After ruling out most common diagnoses, a significant but variable proportion of patients remain without any diagnosis (2–25%) [1, 6, 7], and a splenectomy for diagnostic purposes may even be performed in cases of non-obvious etiology reaching up to 12% of patients [1]. A French-based study focusing on a limited number of splenectomies performed for diagnostic purposes underlines its postoperative value in the diagnosis of lymphoid malignancies [11]. However, in such cases, determining a diagnosis before resorting to splenectomy is certainly a challenge as delayed diagnosis is detrimental to the progression of certain diseases. Splenectomy, however, exposes patients to postoperative complication rates varying from 20 to 52% (postoperative mortality rates were around 1.4–1.8%) [12, 13, 14] as well as the aggravation of underlying diseases, such as Gaucher disease (GD), thalassemia, Hodgkin lymphoma, or primitive myelofibrosis.

Notably, studying the prevalence of etiologies in patients with unexplained SM would help the medical community prioritize investigations toward identifying the causes. To the best of the authors’ knowledge, there are no published data focusing on the etiology of unexplained SM. The prevalence of these rarer etiologies in patients with unexplained SM is not yet known. In a more in-depth stage of etiology research, it is logical to incorporate the search for rare diseases, such as lysosomal storage diseases (LSDs). LSDs are heterogeneous, inherited, metabolic diseases characterized by the abnormal accumulation of substrates in the cells of various organs due to enzyme dysfunctions [15, 16]. GD, one of the most prevalent LSDs, is caused by a deficiency of glucocerebrosidase, which occurs due to GBA gene mutation [15]. Diagnosis of GD by testing glucocerebrosidase activity is the gold standard, but the rarity and non-specificity of some phenotypic aspects of the heterogeneous nature of GD impede the diagnostic approach and may lead to mis- or underdiagnosis. Patients then encounter inappropriate procedures and severe complications, including irreversible bone disease (e.g., osteonecrosis and pathological fractures). Therefore, early diagnosis before the occurrence of irreversible complications underpins the management guidelines of GD, can minimize the emotional and financial burden encountered by patients, and thus, improve the quality of life of these patients [17–19]. This is to be considered for all the rarer etiologies of unexplained SM. In this context, the SplenoMegaly Study (SMS) aimed at estimating the prevalence of GD and other etiologies in patients presenting with unexplained SM after exclusion of first intention-diagnoses (e.g., portal hypertension, hematological malignancy, hemolytic anemia, and infection) based on basic physical examination, patient interview, and routine biological exams (e.g., full blood count, liver enzymes, and reticulocyte count). Additionally, the study aimed to describe the diagnostic tests performed and the most frequent associations observed.

## Methods

### Study design

The SMS was a prospective, observational, multicenter, longitudinal study conducted across 87 active centers in France. The study was approved by the ethics committee (CPP Ouest IV, France) and performed in accordance with the Declaration of Helsinki. Patients or their legal representatives, if applicable, signed an informed consent form prior to participating in the study.

### Patient selection and procedures

Patients who consented to participate in the study were recruited between September 2015 to April 2020 based on the following inclusion criteria: age ≥ 15 years, referred to a hematology or internal medicine department, and presenting with an unexplained SM confirmed by an ultrasound or CT scan with a maximal spleen length ≥ 13 cm. Patients with spleen size ≥ 18 cm were defined under massive SM. Unexplained SM was considered when a first clinical workup based on patient interview, physical examination, and routine biological tests (including hemogram and reticulocyte count, clotting test, liver enzymes, bilirubin, protein electrophoresis, erythrocyte sedimentation rate [ESR], and C-reactive protein [CRP]) was negative or inconclusive for the physicians to reach the underlying disease. Patients with portal hypertension, hemolytic anemia, hematological malignancy, or known diagnosis of GD based on clinical examination, patient interview, and previous initial biological tests were excluded. Furthermore, a center could not enroll > 10% of the total study population to avoid overrepresentation of certain etiologies.

After the first visit, the last visit for each patient took place when an etiology was identified or 18 months after inclusion, whichever occurred first. All examinations and tests conducted for diagnostic purposes were under each physician’s responsibility. Since there is no clinical gold standard for the precise definition of SM, the SplenoCalc^®^ application (published after the start of the study) was used retrospectively, adjusting for patient’s sex and height [20]. 

### Endpoints and assessments

The primary endpoint of the study was to determine the percentage of patients with GD among those with unexplained SM. GD diagnosis was based on peripheral blood leukocyte β-glucocerebrosidase enzyme activity of < 30% of the normal value. Secondary endpoints included the percentage of patients with etiologies other than GD. This included any disease that could be considered in the differential diagnosis of unexplained SM, such as infection, hematological, congestive, inflammatory, neoplastic, infiltrative, and benign tumors, immune and iron deficiencies, and other miscellaneous rare causes. The percentage of patients with each etiology and the percentage of patients with an unknown diagnosis at the end of the study were estimated. The exploratory endpoint included the percentage of patients presenting with certain symptoms, including thrombocytopenia (platelet count < 150 G/L), anemia (below the sex-adjusted normal range), a history of bone pain, monoclonal or polyclonal gammopathy, and cholelithiasis.

### Statistics

Statistical analyses were performed by International Clinical Trials Association Project Management (ICTA PM) using SAS^®^ software (version 9.4; SAS Institute, NC, USA). The sample size was determined in terms of precision (half-length of 95% confidence interval [CI]) to describe the expected percentage of patients diagnosed with rarest diseases, such as GD. Based on literature and medical health information databases, the potential number of patients in the concerned population was estimated between 5,000 and 10,000 based on the PMSI database [21]. With an expected rate of GD diagnosis at 4% and a precision of 1.6% in the concerned population, 500 patients were required to be enrolled in the study.

Univariate and multivariate logistic regressions were performed to identify predictive factors for obtaining a diagnosis and each of the identified etiologies. Univariate regressions were performed (for diagnostic categories with ≥ 25 patients). Otherwise, multivariate logistic regressions were performed when sample size was sufficient (i.e., > 45 patients). For multivariate logistic regressions, a stepwise procedure with a 10% threshold for the input and a 10% threshold for the output of the variables was used to select the variables in the final model [22]. Quantitative variables were compared using Mann–Whitney–Wilcoxon test or Student’s *t*-test. Qualitative variables were compared using Chi^2^ test or Fisher’s exact test. All tests were two-sided and type 1 error was set at 5%.

## Results

### Study populations and baseline characteristics

**Fig. 1:**
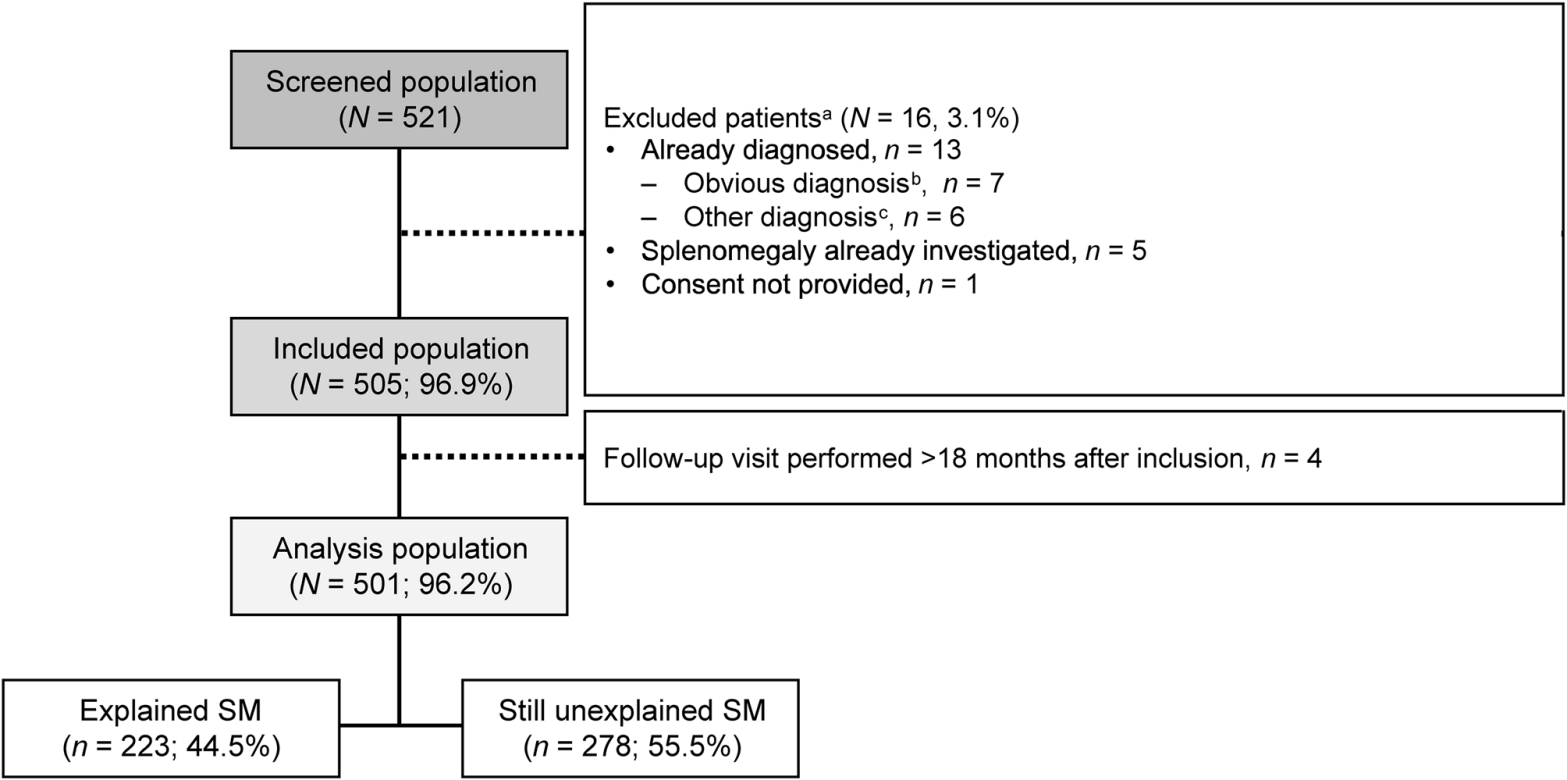
Disposition of patients. ^a^Patients could have several reasons of non-inclusion. ^b^Obvious diagnoses included portal hypertension, hemolytic anemia, or hematological malignancy. ^c^Including Gaucher disease, *n* = 1; unknown origin *n* = 5. SM, splenomegaly

From September 2015 to April 2020, 521 patients were screened in 87 centers. Active investigators were internists (52.3%), hematologists (45.5%), nephrologists (1.1%), and general practitioners (1.1%) who were practicing mainly in general hospitals (57.5%) distributed throughout Metropolitan France. A total of 505 patients met all eligibility criteria and were enrolled in the study, of which 501 continued follow-up visits up to 18 months after inclusion (Fig. 1). The median (range) number of included patients per site was 3.0 (1–50).

At inclusion, the total population (*N* = 501, median [range] 51.0 [16–94] years) comprised 61.5% of male patients, with 23.4% of patients having a body mass index > 30 kg/m². The median (range) spleen length was 15.0 [13; 30] cm, with massive SM (spleen length ≥ 18 cm) observed in 17.6% of patients and heterogeneous SM structure in 11.2% of patients (Table 1). The relevant medical/surgical history per system organ class according to diagnosis at the follow-up visit is presented in Additional file 1, Supplementary Table 1. A previous history of SM was found in 72 patients (14.7%) and was associated with unexplained SM when compared to explained SM (18.5% vs. 10.1%, *p* = .010).

**Table 1:** Baseline demographics and clinical characteristics stratified by group

Category	Parameter	Explained SM (*n* = 223)	Still unexplained SM (*n* = 278)	*p*-value	Total (*N* = 501)
**Females**	*n* (%)	93 (41.7)	100 (36.0)	0.190	193 (38.5)
**Males**	*n* (%)	130 (58.3)	178 (64.0)		308 (61.5)
**Age at inclusion**,** years**	Mean (SD)	55.5 (18.9)	47.6 (19.0)	< 0.001	51.1 (19.4)
	Median	60.0	45.0		51.0
	Min; Max	16; 94	17; 91		16; 94
**BMI**,** kg/m²**	*N*	217	262	0.194	479
< 18.5	*n* (%)	12 (5.5)	7 (2.7)		19 (4.0)
[18.5–30]	*n* (%)	159 (73.3)	189 (72.1)		348 (72.7)
> 30	*n* (%)	46 (21.2)	66 (25.2)		112 (23.4)
**Spleen length**,** cm**	*N*	223	278	<.001^a^	
	Mean (SD)	16.4 (3.1)	15.3 (2.0)		15.8 (2.6)
	Median	15.7	15.0		15.0
	Min; Max	13; 30	13; 25		13; 30
**Spleen length**,** cm**				<.001^b^	
< 18	*n* (%)	164 (73.5)	249 (89.6)		413 (82.4)
≥ 18	*n* (%)	59 (26.5)	29 (10.4)		88 (17.6)
**SM according to SplenoCalc** ^c, d^	*N*	219	268	.411^b^	487
Confirmed	*n* (%)	191 (87.2)	241 (89.9)		432 (88.7)
Not confirmed	*n* (%)	12 (5.5)	15 (5.6)		27 (5.5)
Size out of range	*n* (%)	16 (7.3)	12 (4.5)		28 (5.7)
**Spleen structure**	*N*	195	235	0.001	430
Homogeneous	*n* (%)	162 (83.1)	220 (93.6)		382 (88.8)
Heterogeneous	*n* (%)	33 (16.9)	15 (6.4)		48 (11.2)

BMI, body mass index; SD, standard deviation; SM, splenomegaly

^a^Wilcoxon–Mann–Whitney test (for quantitative variables)

^b^Pearson’s Chi² test (for qualitative variable) was conducted with a significance level of 5%

^c^SplenoCalc^®^ application, which calculates approximate percentiles of an individual’s spleen size

^d^Height was not available for 14 patients

According to SplenoCalc^®^ application, SM was confirmed in 432 of 487 (88.7%) patients and invalidated in 5.5% without any statistical difference between patients with or without a final diagnosis (*p =*.411). Of note, it could not be determined in 5.7% of patients because their height was outside the application standards.

A precise diagnosis was reached in 223 (44.5%) of patients, with a median (min, max) duration between inclusion and diagnosis of 4.9 (0, 17) months. Compared to patients with still unexplained SM, patients who received a diagnosis were significantly older (median age: 60.0 vs. 45.0 years, *p* <.001), had more often a massive SM (spleen size ≥ 18 cm, 26.5% vs. 10.4%, *p* <.001), and had a heterogeneous spleen structure (16.9% vs. 6.4%, *p* = .001). In addition, patients with explained SM displayed more frequently altered biological test results (Additional file 1, Supplementary Table 2), including lower platelet count; a greater proportion of patients presented with thrombocytopenia (platelet count < 100 G/L in 23.3% vs. 11.9% of patients, *p* = .001), lower median hemoglobin levels (12.5 g/dL vs. 14.0 g/dL, *p <*.001; abnormal in 61.4% vs. 33.5% of patients), lower leukocyte levels (abnormal ≤ 1.5 G/L in 29.1% vs. 18.3% of patients, *p =*.004), higher median CRP levels (5.0 mg/L vs. 3.5 mg/L, *p* = .005; abnormal [≥ 6 mg/L] in 46.9% vs. 33.2% of patients, *p* = .003), and higher median gamma-glutamyl transferase (γ-GT) levels (36.0 UI/L vs. 29.0 UI/L, *p* = .007; abnormal in 35.3% vs. 25.6% of patients, *p* = .022). No differences were found in other liver function parameters (aspartate aminotransferase and alanine aminotransferase).

**Fig. 2:**
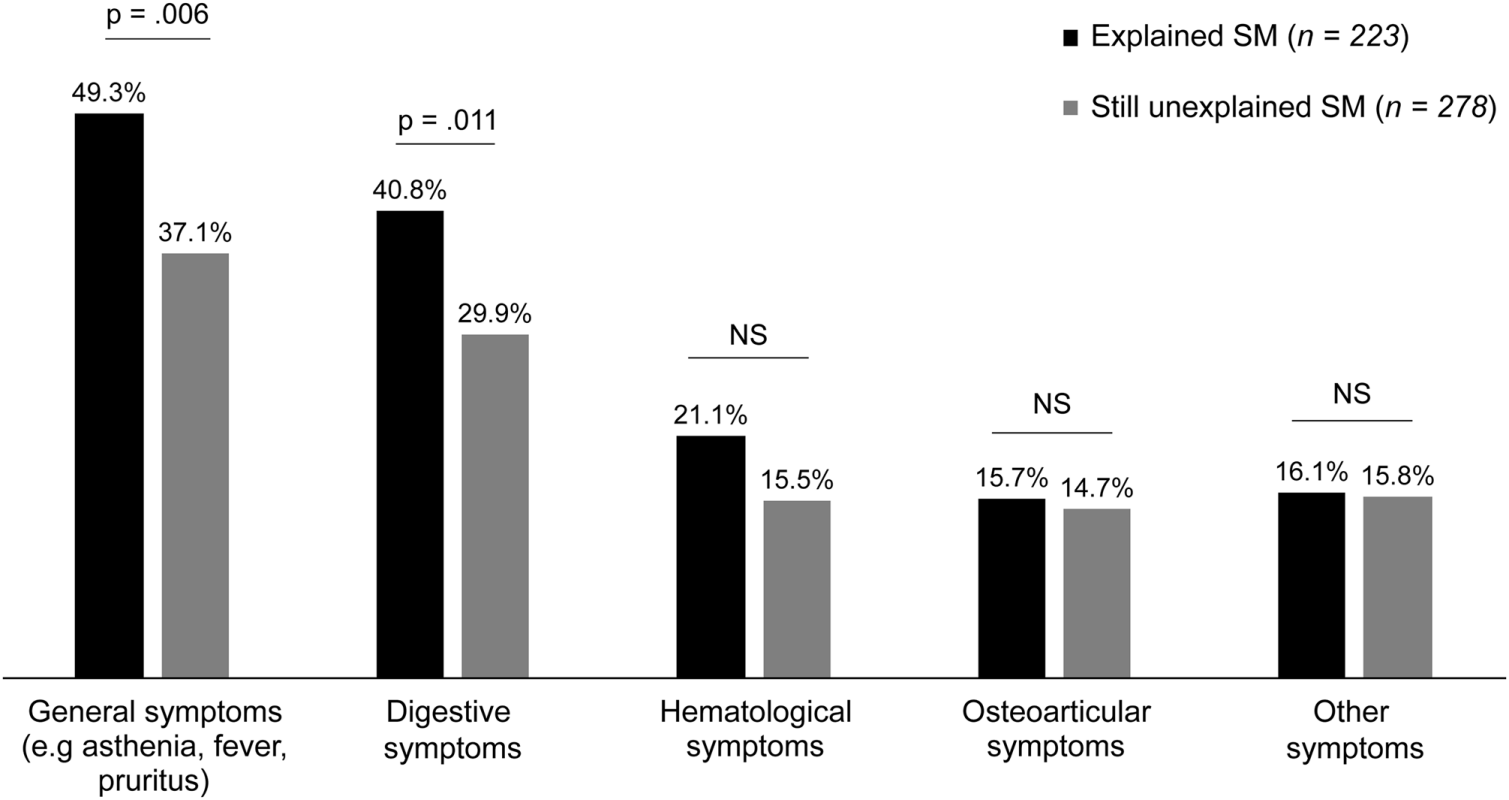
Patient symptomatology stratified by group (*N* = 501). NS, non-significant; SM, splenomegaly. Pearson’s Chi² test was conducted with a significance level of 5%

Patients with an explained SM showed significantly more general symptoms, specifically asthenia, fever, pruritus, and others, and digestive symptoms at inclusion than patients with still unexplained SM (Fig. 2). Fever is part of the category of general symptoms, along with fatigue and pruritus. Hematological symptoms corresponded to purpura, petechiae, hemorrhage, lymphadenopathy, and other related conditions. Osteoarticular or other symptoms were not significantly different between the study groups.

### Prevalence of identified etiologies

**Fig. 3:**
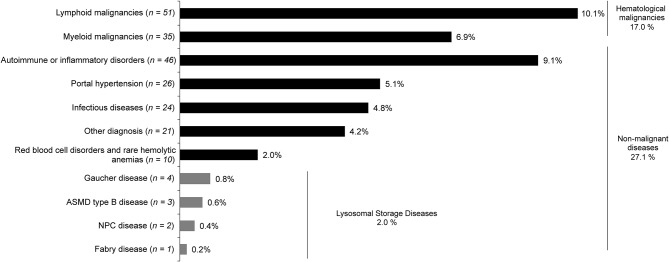
Prevalence of identified etiologies for SM (*N* = 505). ASMD, acid sphingomyelinase deficiency; NPC, Niemann–Pick type C; SM, splenomegaly

The etiologies identified were divided into hematological malignancies and non-malignant disease with a prevalence of 17% and 27.1%, respectively. The most prevalent ones were lymphoid malignancies, immunological disorders, myeloid malignancies, portal hypertension, infectious diseases, and red blood cell disorders (Fig. 3; Additional file 1, Supplementary Table 3). None of the patients had cholelithiasis.

**Fig. 4:**
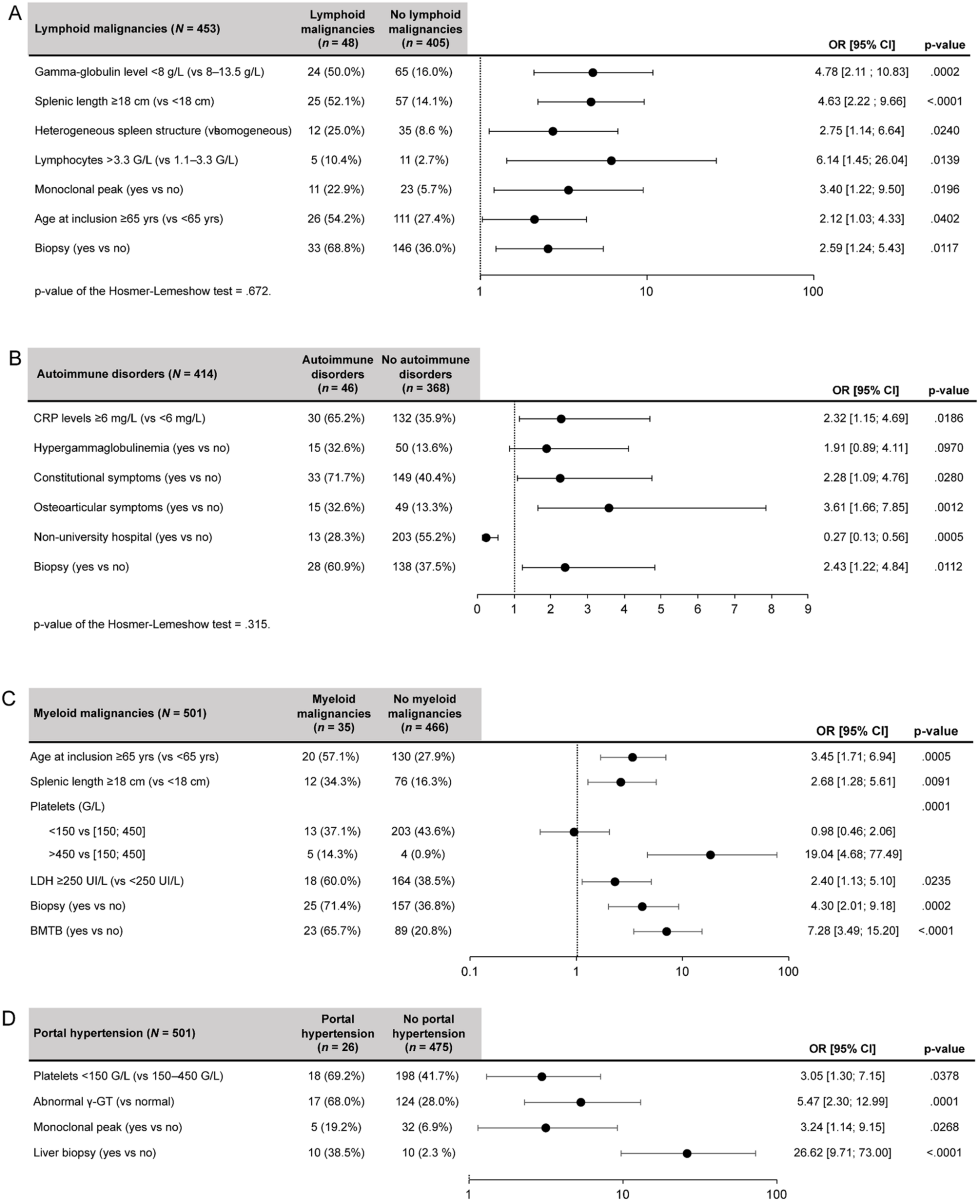
Predictive factors for lymphoid malignancies^a^ (**A**), autoimmune disorders^a^ (**B**), myeloid malignancies^b^ (**C**), and portal hypertension^b^ (**D**). ^a^All predictive factors obtained with the multiple regression analyses are presented. Independent variables with *p* <.01 in the univariate regression analysis were introduced in the model. The number of observations used was lower than the sample size because of missing data for some variables. ^b^Univariate regression analysis with predictive factors with *p* ≤.05 presented. The observed number of patients with myeloid malignancies and portal hypertension was too low to proceed to a multiple regression analysis through the numerous predictive factors identified. BMTB, bone marrow trephine biopsy; CI, confidence interval; CRP, C-reactive protein; γ-GT, gamma-glutamyl transferase; LDH, lactate dehydrogenase; OR, odds ratio; vs., versus; yrs, years

Ten patients were identified with LSDs (prevalence, 2.0%): 4 patients with GD, 3 patients with acid sphingomyelinase deficiency (ASMD) type B disease, 2 patients with Niemann–Pick type C disease, and 1 patient with Fabry disease. The prevalence of GD was 0.8% of the total included patients (*n* = 505), corresponding to 4 patients (2 male and 2 female patients; Additional file 1, Supplementary Table 4). All patients had thrombocytopenia, with platelet levels < 150 G/L (from 77 G/L to 101 G/L); Case 1 described a 35-year-old male presenting with bone pain, Case 2 described a 27-year-old female presenting with mild anemia (hemoglobin: 11.0 g/dL), Case 3 described a 17-year-old female with no other relevant clinical symptoms than thrombocytopenia, and Case 4 described a 62-year-old male with polyclonal gammopathy.

### Tests and examinations

For patients with explained SM, investigators could report a maximum of 3 tests that led to the diagnosis; this was reported for 86.1% of patients. The most performed examinations for explained SM were various organ biopsies (48.0% of patients), imaging (38.1% of patients), and flow cytometry (18.4% of patients; Additional file 1, Supplementary Table 5). Bone marrow trephine biopsy (BMTB) was the most frequent type of biopsy performed in 52.3% of patients with explained SM. In total, 112 patients had BMTB, 56 had an explained SM and 56 had no diagnosis. 25.1% of patient with explained SM had BMTB. Among patients with explained SM, the most frequently performed association of tests was BMTB and molecular biology (performed in 6.3% of patients (*n* = 14), including 9 patients who only had these 2 examinations). The other most frequent test associations were BMTB with flow cytometry or bone marrow examination (each performed in 5.8% of patients).

For patients with still unexplained SM, all tests performed were considered to establish a diagnosis. In consequence, the median (range) number of tests and examinations performed in those patients was 13.0 (0–51) per patient. The most frequent test categories were imaging (77.8%), biology (91.8%), serology (88.2%), and autoimmunity (69.0%) (Additional file 1, Supplementary Table 5). Thus, as the method of collection was not similar, no comparison could be drawn between those patients and patients with explained SM.

In addition, most patients (80.2%) underwent a β-glucocerebrosidase activity assay to test for GD, and the prevalence of type 1 GD was 1.0% in those patients (*n* = 402). However, among patients with still unexplained SM (*n* = 278), 22.3% were not tested for GD. Diagnostic splenectomy was performed for 6 (2.5%) and 16 (7.2%) patients in the still unexplained SM and explained SM subgroups, respectively, with at least one relevant medical/surgical history in 4/6 (66.6%) patients with still unexplained SM and 12/16 (75.0%) patients with explained SM. A history of metabolism and nutrition disorders and nervous system disorders was reported for 2 (33.3%) patients in the still unexplained SM group who underwent diagnostic splenectomy.

### Predictive and associated factors to splenomegaly diagnostic categories

The following predictive factors were associated with higher odds of being diagnosed with lymphoid malignancies (compared to reference categories): hypogammaglobulinemia, massive SM, heterogeneous spleen structure, lymphocytes, presence of a monoclonal peak, older age at inclusion, and biopsy exploration (Fig. 4 A).

High CRP levels, constitutional, and osteoarticular symptoms, and biopsy exploration, were significantly associated with higher odds of having an accurate diagnosis of autoimmune or inflammatory disorders than the reference categories (Fig. 4 B). On the contrary, patients treated in institutions other than university hospitals were significantly less likely to be diagnosed with autoimmune or inflammatory disorders.

Older age at inclusion, massive SM, high platelets counts, and lactate dehydrogenase levels, biopsy exploration, and BMTB exploration were all significantly associated with higher odds of being diagnosed with myeloid malignancies (compared to the reference categories; Fig. 4C).

Low platelet levels, abnormal γ-GT levels, presence of a monoclonal peak, and liver biopsy exploration were all significantly associated with higher odds of having a diagnosis of portal hypertension (compared to the reference categories; Fig. 4D).

## Discussion

The SMS is the first real-life observational study to investigate the etiologies of “unexplained” SM and confirm the diagnostic challenges these patients face. During the study, a significant proportion (44.5%) of patients with unexplained SM, who had undergone investigations for varying durations prior to the study, ultimately received a diagnosis through further investigations. However, 55.5% of 501 patients remained without diagnosis within 18-month follow-up. Several explanations can be proposed. First, the observational methodology prevented a predefined diagnosis approach such that physicians were not directed in their workup and patients could refuse some invasive examinations (e.g., BTMB). We found that 5.6% of these patients with a maximal spleen length ≥ 13 cm was not true SM, by using the height and sex weighting of SplenoCalc^®^ application, but invalidated SM in a similar proportion in subgroup with or without a final diagnosis as the calculation limit designates women < 155 cm or ≥ 180 cm in height as well as men < 160 cm in height, as having an out-of-bound size that does not allow the determination of splenomegaly. Moreover, the follow-up time was limited, and some additional cases were diagnosed after the end of the study. Finally, patients who had SM of unknown origin and did not feel unwell were younger, with few clinical symptoms and biological abnormalities. Conversely, older patients with massive SM or heterogenous structure were more likely to be diagnosed.

Extensive medical workup is challenging for physicians as many signs and symptoms are common to various conditions. This long medical course was confirmed by data observed in this study, considering the variety and high number of tests and examinations performed. Indeed, undiagnosed patients underwent an average of 14 tests and examinations. The most frequent tests were related to imaging, biology, and serology, both for patients with explained and unexplained SM. However, notably, biopsies were more frequently performed in diagnosed than undiagnosed patients, with BMTB being the most frequent and relevant. Only 9 imaging-guided percutaneous spleen biopsies were reported compared to 16 hepatic ones. This low splenic biopsy rate reflects the French practice indicating concern for hemorrhagic risk [23, 24]. Although a meta-analysis found a high performance for diagnosis and a quite low hemorrhagic complication rate of approximately 1.3% compared to kidney biopsies [25]. Diagnostic splenectomy was performed in 22 (4.8%) patients, of whom 16 contributed directly to the diagnosis, which was mainly lymphoid malignancies (75.0% of them). Previous studies reported that among patients who underwent a splenectomy for any reason, diagnostic splenectomy was performed in 9.2% [11] to 12% [1] of patients with SM. The lower observed splenectomy rate in the current study could be explained by the modification of clinical practice in France where few surgical actions are performed for diagnostic purposes due to safety reasons [23, 24]. 

In SMS, the main etiologies of SM were non-malignant diseases (combining autoimmune or inflammatory disorders, portal hypertension, infectious diseases, and marginally LSDs) with a total prevalence of 27.1%, while hematological disorders have usually been reported as the main cause of SM [1, 7, 11]. Even if infectious diseases are much less frequent than in the past decade (they represented 16–36% in the American study), they are still present (4.8%) and need to be ruled out, especially tuberculosis [1]. It is noteworthy that various red blood cell disorders, mainly inherited, come to light as isolated SM (2%); thus, hemolysis has to be carefully searched, even without anemia. Among hematological neoplasms, lymphomas were the leading cause with splenic marginal zone lymphoma (18/49) being the most prominent B-cell-derived lymphoma (Additional file 1, Supplementary Table 6). In myeloid neoplasms, primary or secondary myelofibrosis is most common (14/35). Indeed, in some cases, the analysis of white blood cells and blood films failed to provide convincing results, and other examinations were warranted, such as chromosomal microarray familial testing, karyotype, next-generation sequencing (NGS), and BMTB.

A total of 10 (2.0%) patients were diagnosed with LSDs, including 4 with GD (representing a prevalence of 0.8%). The prevalence of GD was lower in SMS study than previously described [26, 27]. Indeed, the expected rate of patients with GD was 4.0%, and in a recent study, the prevalence of GD was reported at 3.3% in patients with SM and/or thrombocytopenia [26]. 

Patients from this cohort were younger than in the current study (mean age, 46.9 years vs. 51.0 years). Above all, the higher GD prevalence in the study from Motta et al. [26] could be explained by the study design, which was in favor of enriching the population with patients with GD: patients needed to have ≥ 1 other sign or symptom of GD (based on Mistry P’s algorithm [28]) in addition to SM or thrombocytopenia, compared to SMS study wherein no specific patients were targeted (all patients with unexplained SM could be included). Nevertheless, this prevalence at the end of SMS study is higher than the incidence of new cases of GD observed in real life by the French MG expert group (in the order of 2–6 per year for the whole of Metropolitan France. However, GD prevalence could be underestimated in the SMS study since some patients with GD could not be included because the β-glucocerebrosidase enzyme activity assay had already been performed before enrollment. Patients with still unexplained SM who may have LSD could also not be excluded at the end of the study as 22.3% of them were not tested for GD or ASMD. This is also the reason these data support that an enzymatic assay for LSD, such as β-glucocerebrosidase and acid sphingomyelinase for GD and Niemann–Pick disease, respectively [29], be systematically performed in patients presenting with unexplained SM. In addition, the descriptive analysis of the 4 patients with GD showed patients with very heterogeneous clinical symptoms and thus, also highlighted the relevance of a systematic screening for GD by appropriate enzymatic tests in patients with SM and thrombocytopenia (platelet levels < 150 G/L) because no clear and specific clinical profile can be drawn for these patients.

Unlike GD and ASMD, where SM is frequent (80% and 60%, respectively), SM is not frequent in Fabry disease (α-galactosidase A congenital deficiency). Nevertheless, SM has been yet described, and the spleen enlargement was due to high blood splenic flow and globotriaosylceramide (Gb3) and Lyso-Gb3 endothelial cell accumulation [30]. 

SM may be consecutive to mild portal hypertension noted in approximately 5% of cases and clinicians may be suspicious of that especially if patients have metabolic syndrome and/or the observed risk factors. Mild thrombocytopenia and high γ-GT levels are intuitive but monoclonal gammopathy seems also associated as it is well-known in cirrhosis. In this setting, the patient may be referred to a hepatologist for more explorations such liver biopsy.

Compared to patients with still unexplained SM after the follow-up period, patients with explained SM were significantly older, with a larger and heterogeneous spleen, more severe symptoms, and altered biological parameters. Therefore, more severe clinical symptoms made it easier for physicians to establish a diagnosis by facilitating diagnostic orientation. This confirms again the difficulty in diagnosing patients with a wide range of symptoms less characteristic of a particular etiology.

Site representativeness was not assessed, but to prevent any selection bias, a large sample of physicians was invited to participate in the study (> 3,700 physicians, representing around 80% of targeted French physicians). Physicians targeted for participation were internists or hematologists considering the possible etiologies associated with SM [21]. In adherence to the protocol, no selection of site was performed according to physicians’ specialty or region of practice; the study was proposed to all responders.

The study is constrained by the limited sample size of patients diagnosed with GD (*n* = 4). It should be noted that univariate and multivariate logistic regression analyses could not be conducted to establish the predictive significance of diagnostic criteria for GD, as defined by Mistry P (including thrombocytopenia, anemia, history of bone pain, early onset of mono- or polyclonal gammopathy in patients < 30 years, along with cholelithiasis) could not be performed [28]. Despite this, a descriptive analysis of the patients revealed that some of the diagnostic criteria, such as young age, were present in all patients except for one who was 62 years old, with all but 1 patient experiencing thrombocytopenia (platelet levels < 100 G/L; 1 patient had a platelet level of 101 G/L), 1 patient having anemia, and 1 patient suffering from bone pain. Polyclonal gammopathy was found in the oldest male patient, and no patients had cholelithiasis. The clinical symptoms of these 4 patients with GD were heterogeneous, underscoring the importance of systematic screening for GD in patients with SM and thrombocytopenia (platelet levels < 150 G/L).

The diagnostic strategy for SM should be optimized; to assist physicians in making a possible diagnosis, a list of potential predictive factors has been identified through exploratory analyses. These results could help physicians to be more attentive to particular symptoms and suggest better management in terms of cost and time, and fewer invasive examinations, leading to relief for patients if adequate treatment can be implemented more quickly. These exploratory predictive factors are concrete elements to help practitioners in their approach when investigating unexplained SM. In addition, the use of new technologies, such as NGS panel, might help physicians reduce diagnostic delay for patients presenting with unexplained SM [31]. Indeed, several targeted sequencing panels have been developed for lymphoid, myeloid, rare blood cell disorders, or LSD [32, 33]. The NGS approach represents a valuable alternative to biochemical assays, mainly because a broader spectrum of diseases can be monitored in one single test in a shorter time.

## Conclusion

In conclusion, patients with an apparent idiopathic SM could reach a precise diagnosis in approximately 44.5% of cases. The use of the SplenoCalc^®^ application published since the start of this study means that certain diagnoses of moderate SM can be ruled out and thus, the calculation should be used more widely.

Patients with explained SM were older and had a larger spleen and altered biological parameters compared to those with SM remaining unexplained at the end of the study. Interestingly, non-malignant diseases were more prevalent compared to hematological malignancies. The prevalence of rare disease was high: 10 patients were diagnosed with LSD, including 4 with GD (prevalence, 0.8%). These data support that an enzymatic assay for LSD should be systematically performed in patients with unexplained SM, especially before splenectomy, particularly because of the risk of aggravating the underlying disease [14, 34]. This study also allowed to define a potential list of predictive factors for the main diagnostic categories of unexplained SM, providing useful new information to optimize the diagnostic strategy of SM.

## Electronic supplementary material

Below is the link to the electronic supplementary material.

Supplementary Material 1

## Data Availability

Data are available at request from the corresponding author.
