# Supplementary material for: SplenoMegaly study (SMS): exploring the etiologies for “unexplained” splenomegalies in the real world
Source: Orphanet J Rare Dis. 2025 Jun 6;20:285. doi: 10.1186/s13023-025-03768-3 (PMC12142850; doi:10.1186/s13023-025-03768-3)
Supplement: Supplementary file 1 — Supplementary Material 1 [file 13023_2025_3768_MOESM1_ESM.docx]

**TABLES**

**Supplementary Table 1: Relevant medical/surgical history per SOC^a,b^ according to diagnosis at the follow-up visit.**

|  | **Still unexplained SM  (*N* = 278)** | **Explained SM  (*N* = 223)** | **Total  (*N* = 501)** |
| --- | --- | --- | --- |
| **At least 1 relevant medical/surgical history, *n* (%)** | 198 (71.2) | 161 (72.2) | 359 (71.7) |
| **Surgical and medical procedures, *n* (%)** | 51 (18.3) | 38 (17.0) | 89 (17.8) |
| **Metabolism and nutrition disorders, *n* (%)** | 42 (15.1) | 37 (16.6) | 79 (15.8) |
| **Vascular disorders, *n* (%)** | 33 (11.9) | 34 (15.2) | 67 (13.4) |
| **Musculoskeletal and connective tissue disorders, *n* (%)** | 25 (9.0) | 26 (11.7) | 51 (10.2) |

MedDRA, Medical Dictionary for Regulatory Affairs; SM, splenomegaly; SOC, system organ class.

^a^SOCs with >10% incidence are reported.

^b^Coding was done from MedDRA dictionary Version 24.0.

**Supplementary Table 2: Baseline biological characteristics stratified by group.**

|  |  | **Still unexplained SM**  **(*N* = 278)** | **Explained SM**  **(*N* = 223)** | ***p-*value^a^** |
| --- | --- | --- | --- | --- |
| **Platelets, G/L** | *N* | 278 | 223 |  |
|  | Median | 166.5 | 159.0 | .099 |
| <100 G/L | *n* (%) | 33 (11.9) | 52 (23.3) | .001 |
| **Hemoglobin, g/dL** | *N* | 278 | 223 |  |
|  | Median | 14.0 | 12.5 | <.001 |
| Abnormal^b^ | *n* (%) | 93 (33.5) | 137 (61.4) | <.001 |
| **Leucocytes, G/L** | *N* | 278 | 223 |  |
|  | Median | 5.7 | 5.4 | .031 |
| Abnormal^c^ | *n* (%) | 51 (18.3) | 65 (29.1) | .004 |
| **Reticulocytes (G/L)** | *N* | 209 | 194 |  |
|  | Median | 75.0 | 71.5 | .619 |
| Abnormal^d^ | *n* (%) | 73 (34.9) | 68 (35.1) | .979 |
| **γ-GT, UI/L** | *N* | 257 | 219 |  |
|  | Median | 29.0 | 36.0 | .007 |
| Abnormal^e^ | *n* (%) | 64 (25.6) | 77 (35.3) | .022 |
| **ASAT, UI/L** | *N* | 269 | 222 |  |
|  | Median | 22.0 | 24.0 | .055 |
| **ALAT, UI/L** | *N* | 270 | 222 |  |
|  | Median | 25.0 | 23.0 | .280 |
| **CRP, mg/L** | *N* | 241 | 211 |  |
|  | Median | 3.5 | 5.0 | .005 |
| Abnormal^f^ | *n* (%) | 80 (33.2) | 99 (46.9) | .003 |
| **Albumin, g/L** | *N* | 273 | 219 |  |
|  | Median | 42.7 | 40.3 | <.001 |
| Abnormal^g^ | *n* (%) | 119 (43.6) | 130 (59.4) | .001 |
| **Alpha 1 globulins, g/L** | *N* | 273 | 217 |  |
|  | Median | 2.9 | 3.3 | <.001 |
| **Alpha 2 globulins, g/L** | *N* | 274 | 218 |  |
|  | Median | 6.5 | 6.2 | .277 |
| **Beta globulins, g/L** | *N* | 274 | 218 |  |
|  | Median | 7.2 | 7.1 | .855 |
| **Gamma globulins, g/L** | *N* | 274 | 218 |  |
|  | Median | 10.8 | 10.8 | .890 |
| Abnormal^h^ | *n* (%) | 104 (38.0) | 108 (49.5) | .010 |
| **Monoclonal peak** | *N* | 274 | 219 |  |
|  | *n* (%) | 15 (5.5) | 22 (10.0) | .056 |
| **Beta-gamma block** | *N* | 274 | 219 |  |
|  | *n* (%) | 4 (1.5) | 1 (0.5) | .388 |
| **Polyclonal hypergamma-globulinemia** | *N* | 274 | 219 |  |
|  | *n* (%) | 36 (13.1) | 38 (17.4) | .193 |

ALAT, alanine aminotransferase; ASAT, aspartate aminotransferase; CRP, C-reactive protein; γ-GT, gamma-glutamyltransferase.

^a^Wilcoxon–Mann–Whitney test (for quantitative variables) and Pearson’s Chi² test (for qualitative variables) was conducted with a significance level of 5%.
^b^Hemoglobin normal range: female [12.1–16.4] g/dL; male [14–17.9] g/dL.

^c^Leucocytes normal range was [1.1–3.3] G/L.

^d^Reticulocytes normal range was [25–85] G/L.

^e^γ-GT normal range was not standardized and was defined by medical laboratories.

^f^CRP normal range was <6 mg/L.

^g^Albumin normal range was [40.2–47.6] g/L.

^h^Gamma globulins normal range was [8–13.5] g/L.

**Supplementary Table 3: Identified etiologies.**

| **Category** | **Subtype** | **Count** |
| --- | --- | --- |
| **Lymphoid Neoplasms  (*N* = 51)** | B-cell derived | 43 |
|  | SMZL | 18 |
|  | B-cell NHL not specified | 10 |
|  | DLBCL | 7 |
|  | MCL | 3 |
|  | FL | 2 |
|  | SLL | 1 |
|  | WM | 1 |
|  | EBV+LPD | 1 |
|  | T/NK-cell derived | 5 |
|  | T-NHL not specified | 3 |
|  | Angioimmunoblastic T-cell lymphoma | 1 |
|  | NK LGL leukemia | 1 |
|  | Hodgkin Lymphoma | 1 |
|  | Not specified | 1 |
| **Myeloid Neoplasms  (*N* = 35)** | Myelofibrosis | 14 |
|  | PMF | 13 |
|  | Post-ET-MF | 1 |
|  | ET | 6 |
|  | MPN-U | 6 |
|  | MDS/MPN | 5 |
|  | MDS | 3 |
|  | PV | 1 |
| **Immunological Disorders (*N* = 47)** | Sarcoidosis | 11 |
|  | SLE | 7 |
|  | Primary immunodeficiencies | 7 |
|  | Hemophagocytic lymphohistiocytosis | 5 |
|  | Sjogren's syndrome | 4 |
|  | Biermer disease | 3 |
|  | Adult onset Still's disease | 3 |
|  | Undifferentiated connective tissue | 2 |
|  | Multiple autoimmune syndrome | 1 |
|  | APS | 1 |
|  | RA | 1 |
|  | RCPA | 1 |
| **Lysosomal Storage Diseases (*N* = 10)** | Gaucher | 4 |
|  | ASMD | 3 |
|  | NPC | 2 |
|  | Fabry | 1 |
| **Portal Hypertension  (*N* = 27)** | NASH | 17 |
|  | Cryptogenic cirrhosis | 3 |
|  | Portal thrombosis | 2 |
|  | Regenerative nodular hyperplasia | 1 |
|  | Primary sclerosing cholangitis | 1 |
|  | Porto-sinusoidal vascular disease | 1 |
|  | Cholestatic liver disease (ABCB11 homozygous mutation) | 1 |
|  | Not specified | 1 |
| **Infectious Etiologies  (*N* = 24)** | Bacterial | 14 |
|  | Tuberculosis | 4 |
|  | Subacute endocarditis | 3 |
|  | Chronic osteomyelitis | 2 |
|  | Syphilis | 1 |
|  | Q fever | 1 |
|  | Pseudomononas septicemia | 1 |
|  | Bartonnela | 1 |
|  | Intravenous drug abuse | 1 |
|  | Viral | 9 |
|  | Not specified | 3 |
|  | EBV | 2 |
|  | CMV | 1 |
|  | CMV and Parvovirus B19 | 1 |
|  | HCV | 1 |
|  | SARS-COV2 | 1 |
|  | Parasitic | 1 |
|  | Leismaniosis | 1 |
| **Red Blood Cell Disorders  (*N* = 10)** | RBC membrane defects | 8 |
|  | Hereditary Spherocytosis | 5 |
|  | Hereditary dehydrated Stomatocytosis | 2 |
|  | Hereditary Elliptocytosis | 1 |
|  | RBC enzyme defects | 1 |
|  | PKD | 1 |
|  | Autoimmune hemolytic anemia | 1 |
| **Primitive splenic diseases (*N* = 14)** | Primitive splenic lesion | 6 |
|  | Hemangioma | 2 |
|  | Hemolymphangioma | 1 |
|  | Angioma | 1 |
|  | Peliosis | 1 |
|  | SANT | 1 |
|  | Accessory spleen | 1 |
|  | Wandering spleen | 1 |
| **Other diagnoses (*N* = 13)** | Congestive | 2 |
|  | FMF | 1 |
|  | Idiopathic hypereosinophilic syndrome | 1 |
|  | Colorectal neoplasia | 1 |
|  | Systemic mastocytosis | 1 |
|  | Hereditary hemochromatosis | 1 |
|  | Chylomicronemia syndrome | 1 |
|  | Others | 5 |

AOSD, Adult-onset Still's disease; APS, Antiphospholipid Syndrome; ASMD, Acid Sphingomyelinase Deficiency; B-NHL, B-cell Non-Hodgkin Lymphoma; CMV, Cytomegalovirus; DLBCL, Diffuse Large B-Cell Lymphoma; EBV, Epstein-Barr Virus; EBV+LPD, Epstein-Barr Virus-associated Lymphoproliferative Disorder; ET, Essential Thrombocythemia; FL, Follicular Lymphoma; FMF, Familial Mediterranean Fever; HCV, Hepatitis C Virus; MCL, Mantle Cell Lymphoma; MDS, Myelodysplastic Syndrome; MDS/MPN, Myelodysplastic/Myeloproliferative Neoplasm; MPN-U, Myeloproliferative Neoplasm-Unclassifiable; NASH, Non-Alcoholic Steatohepatitis; NK LGL leukemia, Natural Killer Large Granular Lymphocyte Leukemia; NPC, Niemann-Pick Disease Type C; PMF, Primary Myelofibrosis; PKD, Pyruvate Kinase Deficiency; PV, Polycythemia Vera; RA, Rheumatoid Arthritis; RCPA, Relapsing Polychondritis with Autoimmune Features; SANT, Sclerosing Angiomatoid Nodular Transformation; SARS-COV2, Severe Acute Respiratory Syndrome Coronavirus 2; SLE, Systemic Lupus Erythematosus; SMZL, Splenic Marginal Zone Lymphoma; SLL, Small Lymphocytic Lymphoma; T-NHL, T-cell Non-Hodgkin Lymphoma; WM, Waldenström Macroglobulinemia.

**Supplementary Table 4: Characteristics of patients with Gaucher disease (*N = 4*).**

| **Sex** | **Age (in years)** | **Thrombocytopenia** | **Platelets (G/L)** | **Anemia** | **Hemoglobin (g/dL)** | **Bone  pain** | **Mono-/polyclonal gammopathy** | **Monoclonal peak** | **Hypergammaglobulinemia** | **Gallstones** | **Splenomegaly according to SplenoCalc**^®^ | **Spleen length  (cm)** |
| --- | --- | --- | --- | --- | --- | --- | --- | --- | --- | --- | --- | --- |
| Male | 35 | Yes | 77 | No | 14.0 | Yes | No | No | No | No | Confirmed | 16.0 |
| Female | 27 | Yes | 93 | Yes | 11.0 | No | No | No | No | No | Confirmed | 18.5 |
| Female | 17 | Yes | 90 | No | 14.0 | No | No | No | No | No | Confirmed | 14.5 |
| Male | 62 | Yes | 101 | No | 14.6 | No | Yes | No | Yes | No | Confirmed | 17.0 |

**Supplementary Table 5: Tests and examinations performed to establish the diagnosis for explained and unexplained SM.**

| **Tests and examinations, *n* (%)** | **Still unexplained SM  (*N* = 278)** | **Explained SM**  **(*N* = 223)** |
| --- | --- | --- |
| Imaging | 186 (77.8) | 85 (38.1) |
| Ultrasound | 108 (58.1) | 22 (25.9) |
| CT scan | 107 (57.5) | 28 (32.9) |
| PET scan | 59 (31.7) | 25 (29.4) |
| Other | 76 (40.9) | 38 (44.7) |
| Biology | 202 (91.8) | 31 (27.9) |
| Myelogram | 94 (39.3) | 31 (13.9) |
| Serology | 194 (88.2) | 18 (16.2) |
| Autoimmunity | 165 (69.0) | 23 (10.3) |
| Biopsy | 75 (31.4) | 107 (48.0) |
| BMTB | 56 (74.7) | 56 (52.3) |
| Spleen | 11 (14.7) | 9 (8.4) |
| Liver | 4 (5.3) | 16 (15.0) |
| Other | 24 (32.0) | 31 (29.0) |
| Flow cytometry | 105 (43.9) | 41 (18.4) |
| Molecular biology | 86 (36.0) | 29 (13.0) |
| SPEP | 67 (30.5) | 10 (9.0) |
| Cytology | 63 (28.6) | 2 (1.8) |
| Genetic test | 33 (13.8) | 25 (11.2) |
| Diagnostic splenectomy | 6 (2.5) | 16 (7.2) |
| Other | 78 (35.5) | 60 (54.1) |

BMTB, bone marrow trephine biopsy; CT, computerized tomography;
PET, positron emission tomography; SM, splenomegaly; SPEP, serum protein electrophoresis.

Four patients did not perform the follow-up visit 18 months after inclusion.

The test collection method is different depending on whether the patient has a diagnosis or not.

1. For patients with still unexplained SM, the data come from the reclassification of the categories of tests and the text fields of the spheres in the CRF
2. The CRF allowed to enter only 3 tests for patients with a diagnosis
3. The 4 patients without test/exam have Gaucher disease

**Supplementary Table 6: Lymphoid neoplasms (*N* = 49).**

| **Type** | ***n*** |
| --- | --- |
| B-cell derived | 43 |
| SMZL | 18 |
| B-cell NHL not specified | 10 |
| DLBCL | 7 |
| MCL | 3 |
| FL | 2 |
| SLL | 1 |
| WM | 1 |
| EBV+LPD | 1 |
| T/NK-cell derived | 5 |
| T-NHL not specified | 3 |
| Angioimmunoblastic T-cell lymphoma | 1 |
| NK LGL leukemia | 1 |
| Hodgkin Lymphoma | 1 |

DLBCL, diffuse large B-cell lymphoma; EBV, Epstein-Barr virus; FL, Follicular lymphoma; LPD, lymphoproliferative disease; LGL, large granular lymphocyte; MCL, mantle cell lymphoma; NHL, non-Hodgkin lymphomas; NK, natural killer; SLL, small lymphocytic lymphoma; SMZL, splenic marginal zone lymphoma; T-NHL, T-cell non-Hodgkin lymphomas; T/NK, T-cell/ natural killer; WM, Waldenström macroglobulinemia.
